# The assessment of microbial infection in children with autism spectrum disorders and genetic folate cycle deficiency

**DOI:** 10.1186/s12887-024-04687-1

**Published:** 2024-03-21

**Authors:** Dmitry Maltsev, Iryna Solonko, Olena Sydorenko

**Affiliations:** Laboratory of Immunology and Molecular Biology, Research Institute of Experimental and Clinical Medicine, O’Bogomolets National Medical University, Kyiv, Ukraine

**Keywords:** Herpesviruses, TTV, Streptococcus, Candida, Borrelia

## Abstract

**Background:**

The results of disparate clinical studies indicate abnormally frequent cases of certain microorganisms in children with autism spectrum disorders (ASD). However, these data require clarification and systematization. The study aims to study the structure of the microbial profile in children with ASD and genetic folate cycle deficiency (GFCD) and consider differences in diagnostic approaches for identifying microorganisms of different types.

**Methods:**

The study analyzed medical data from 240 children (187 boys and 63 girls) with GFCD aged 2 to 9 years. The children had clinical manifestations of ASD (the study group, SG). The control group (CG) included 53 clinically healthy children (37 boys and 16 girls) of the same age but without GFCD. Both groups of children were tested on active herpetic infections (HSV-1/2, VZV, EBV, CMV, HHV-6, HHV-7, HHV-8), ТТV, Streptococcus pyogenes, Candida albicans, Borrelia burgdorferi, Mycoplasma pneumoniae, Chlamydia pneumoniae, Yersinia enterocolitica, Toxoplasma gondii, congenital CMV neuroinfection and postnatal HSV-1/2 encephalitis. The testing used diagnostic methods specified in PubMed-indexed studies.

**Results:**

In the SG, TTV was found in 196 children (82%), HHV-7 – in 172 (72%), HHV-6 – in 162 (68%), EBV – in 153 (64%), Streptococcus pyogenes – in 127 (53%), Candida albicans – in 116 (48%), Borrelia – in 107 (45%), Mycoplasma pneumoniae – in 94 (39%), Chlamydia pneumoniae – in 85 (35%), Yersinia entеrocolitica – in 71 (30%), Toxoplasma gondii – in 54 (23%), congenital CMV neuroinfection – in 26 (11%), and postnatal HSV-1/2 encephalitis – in 11 children (5% of cases) (*p* < p_0.05_; Z < Z_0.05_). In the SG, there was a higher microbial load in older children (*p* < p_0.05_; Z < Z_0.05_). No gender differences were found.

**Conclusions:**

The study described and characterized a specific abnormal microbial spectrum with a predominance of viral opportunistic agents in children with ASD associated with GFCD.

## Background

The results obtained by scientific research of recent decades highlight the importance of immunological factors in the pathogenesis of autism spectrum disorders (ASD) [1]. These studies draw closer attention to the role and place of microbial factors potentially associated with disorders of immune status. These factors can significantly impact the severity of ASD and comorbid pathology in children [2].

Firstly, data from three recent systematic reviews and meta-analyses of randomized controlled clinical trials indicate a link between genetic folate cycle deficiency (GFCD) and ASD in children [3–5]. GFCD can have a direct influence on ASD risk through the phenomenon of hyperhomocysteinemia and other biochemical disorders with neurotoxic effects and indirect mechanisms by affecting epigenetic regulation gene expression in the nervous and immune systems. Secondly, research has demonstrated the immunomodulatory properties of folic acid – a key metabolite, involved in the folate cycle [6]. GFCD and associated disorders of folic acid metabolism, such as vitamin deficiency [7] or cell overload with non-metabolized folate [8], cause states of immunosuppression and immune dysregulation in both experimental animals and humans, including influences on T-lymphocytes, NK-cells, neutrophils, and antibody production. Thirdly, a recent review of controlled clinical trials by Enbescu et al. deepened the modern understanding of various disorders of innate and adaptive immunity in children with ASD [9]. The results indicated reduced immunoresistance and associated immune dysregulation as a characteristic feature of ASD.

It seems likely that the presence of immune dysfunction in ASD implies a decrease in a child’s resistance to microbial factors. Binstock [10] was the first to selectively identify the reduced immunoresistance in children with ASD. The author described a special subgroup of patients with intramonocytic infectious agents, in particular, measles virus, cytomegalovirus, human herpesvirus 6, and Yersinia enterocolitica [10]. The results of numerous clinical studies further confirmed Binstock’s idea about the unequal decrease in resistance to various microorganisms in children with ASD. Research accumulated data on a narrow list of microorganisms with conditionally pathogenic and opportunistic properties, which are typical for patients suffering from ASD. These infectious agents include herpesviruses of various types [11–13], Beta-hemolytic Group A Streptococcus [14], Вorrelia burgdorferi [15], Yersinia enteroсolitica [10], Candida albicans [16], Mycoplasma and Chlamydia pneumoniae [11], and Toxoplasma gondii [17]. At the same time, scientists have not studied infection with some other opportunistic pathogens common in the human population, such as TTV [18], in children with ASD.

There is a need to clarify a specific list of microorganisms that more frequently occur in children with ASD than in the general pediatric population. This list could shed light on the features of resistance to microbial agents and related microbe-induced immune-dependent mechanisms of CNS (central nervous system) disorders, which currently include infectious [2], inflammatory [1], autoimmune [9], and allergic [19]. Microbial agents can directly affect the development of these immune-dependent lesions, such as in cases of the ASD phenotype after an episode of HSV-1-induced temporal necrotic-hemorrhagic encephalitis [2]. As for their indirect role, microbial agents can act as a trigger for immune-dependent complications involved in the pathogenesis of encephalopathy in ASD. An example is autoimmune anti-NMDA (N-methyl-D-aspartate) receptor encephalitis after an episode of reactivated HHV-7 infection [20].

The actual scope of the microbial profile in children with ASD is currently unknown. However, it is hardly limited to the above list of infectious agents. There is no precisely defined structure of infection with the most typical microbes in ASD, including the specific influence of various microbial agents. The latter has a different frequency of occurrence in children with ASD. It is also necessary to study differences between the most informative diagnostic approaches for detecting microorganisms in children with ASD. These microorganisms vary regarding biological properties and parasitization programs in the human body. Data on the age or gender features of the microbial profile are unavailable. The relevance and novelty of this study are the elucidation of these important additional data. This study can allow for the rational redistribution of efforts in further controlled clinical trials to the highest priority microorganisms. Effective diagnostic and therapeutic algorithms for microbial immune-dependent mechanisms of cerebral disorders in children with ASD are currently actively studied. The findings can foster the implementation and development of these algorithms.

### The aim of the study

The study aims to investigate the microbial profile in children with ASD associated with GFCD. The paper also considers differences in diagnostic approaches to the detection of different microorganisms. This data allows for further study of the relationships between biochemical, immunological, microbiological, and immunopathological factors in immune-dependent mechanisms of encephalopathy development in children with ASD.

### Research objectives

1. To demonstrate the proportion of infections caused by various microorganisms to determine the overall structure of the microbial profile in children with ASD associated with GFCD.
2. To show differences between the microbial profiles of participating groups based on gender, age of children, and taxonomic groups of microorganisms to identify signs of microbial infection in children with ASD associated with GFCD compared to healthy peers.
3. Based on the obtained data, to determine the appropriate directions for further research into biochemical, immunological, microbiological, and immunopathological characteristics among children with ASD associated with GDFC; deepen the understanding of the immune-dependent mechanisms of encephalopathy development in ASD.

## Materials and methods

To achieve the research goal, the study retrospectively analyzed the medical data on 240 children (187 boys and 63 girls) aged 2 to 9 years with GFCD and clinical manifestations of ASD. All of them were patients of the specialized neuroimmunological Vivere Clinic (registration dossier No. 10/2212-M dated 22.12.2018, Kyiv, Ukraine). The processes of obtaining data for research and processing of the materials followed agreement No. 150,221 dated 15.02.2021, and the conclusion of the commission of Bioethical expertise (Protocol No. 140 dated 21.12.2020 of the Bogomolets National Medical University). Child psychiatrists carried out the clinical diagnosis of ASD according to the criteria of DSM-IV-TR (Diagnostic and Statistical Manual of Mental Disorders) and ICD-10 (The International Statistical Classification of Diseases and Related Health Problems).

**Fig. 1:**
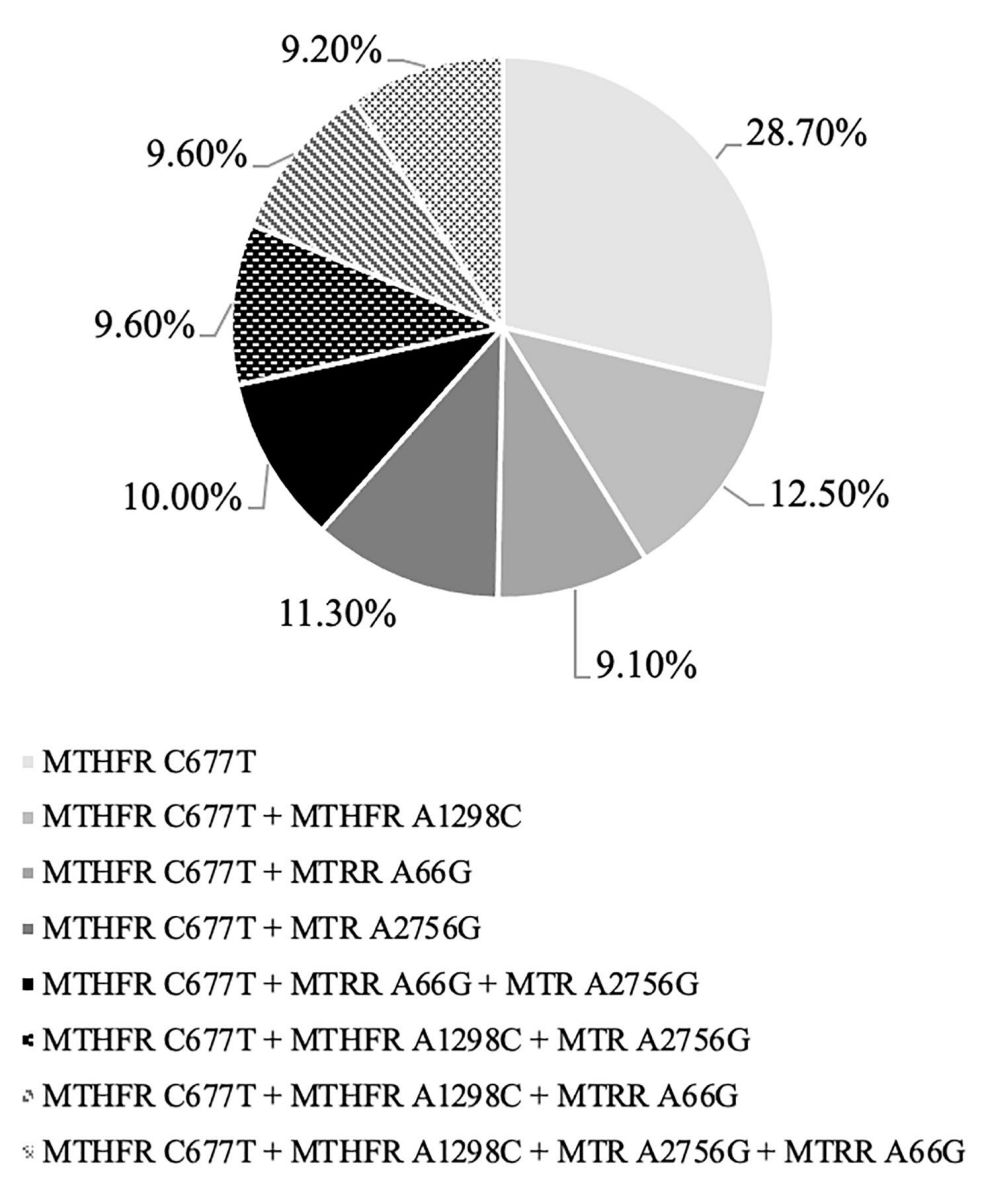
The SG structure based on the results of a genetic GFCD test (*n* = 240)

The study utilized restriction PCR to determine pathogenic polymorphic variants of folate cycle genes based on the detection of MTHFR C677T nucleotide substitution in monoform (69 patients, 28.7%), as well as in combination with other nucleotide substitutions – MTHFR A1298C, MTRR A66G and/or MTR A2756G (171 people, 71.3%). These individuals formed the study group (SG). The genome, which included double pathological nucleotide substitutions MTHFR C677T + MTHFR A1298C was observed in 30 (12.5%) children from the SG, MTHFR C677T + MTRR A66G – in 22 (9.1%), and MTHFR C677T + MTR A2756G – in 27 (11.3% of cases). The genome containing triple pathological nucleotide substitutions MTHFR C677T + MTRR A66G + MTR A2756G occurred in 24 children (10,0%). MTHFR C677T + MTHFR A1298C + MTR A2756G and MTHFR C677T + MTHFR A1298C + MTRR A66G were found in 23 (9,6% of cases for each) children in the SG. Finally, the genome containing all four studied pathogenic nucleotide substitutions, MTHFR C677T + MTHFR A1298C + MTR A2756G + MTRR A66G, was identified in 22 (9.2% of cases) children in the SG (Fig. 1).

The control group (CG) included 53 clinically healthy children (37 boys and 16 girls) of the same age without GFCD. Thus, the comparison of these observation groups demonstrated the differences between the processes of infecting by the studied microorganisms among children with ASD associated with GFCD and their healthy peers.

**Fig. 2:**
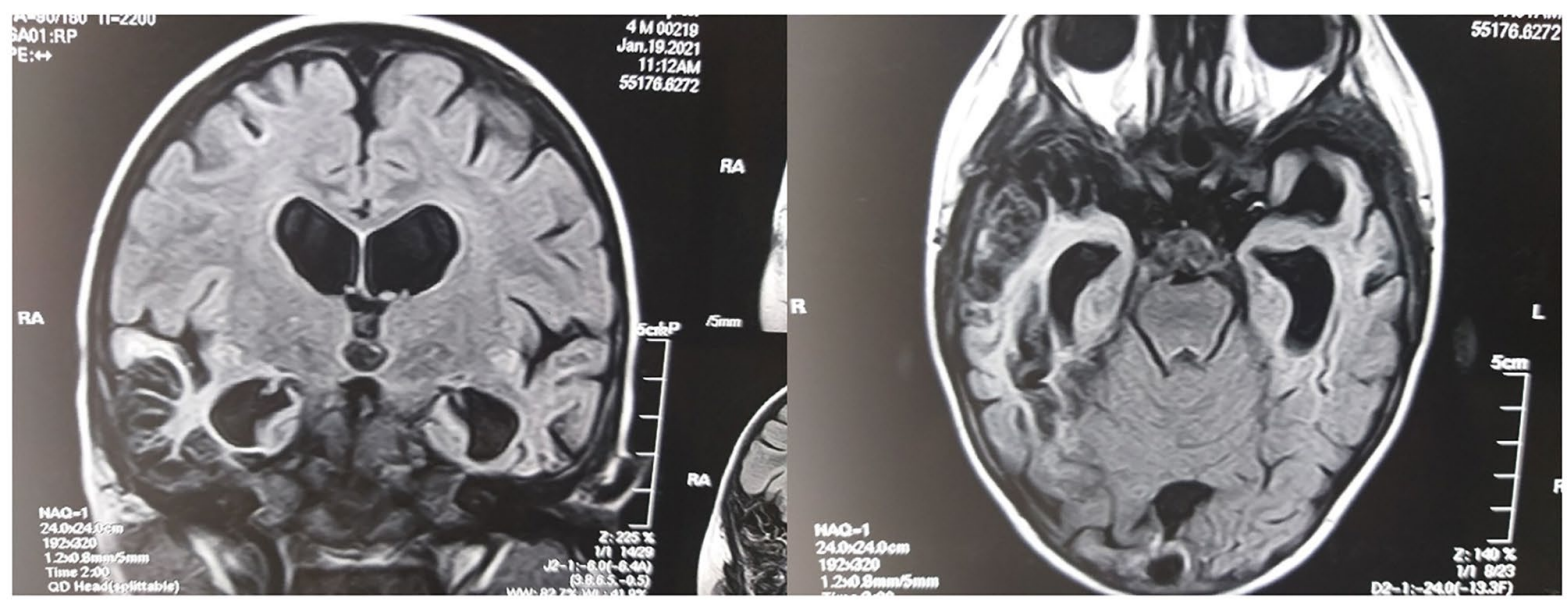
The MRI of the brain in FLAIR mode in coronary (left) and axial (right) projections in a child with ASD associated with GFCD. There are signs of bilateral atrophy and cystic gliosis changes in the temporal lobes of the cerebral hemispheres after bilateral temporal necrotic-hemorrhagic HSV-1-etiology encephalitis (the authors’ observation)

A special paraclinical laboratory microbiological diagnostic examination of children of the observation groups followed current ideas about microbial infection in patients with ASD. Thus, the diagnostic tests of reactivated herpes virus infections (*HSV-1/2, VZV, EBV, CMV, HHV-6, HHV-7, HHV-8*) and *TTV* infections utilized the PCR of white blood cells (The Neurobiochemistry Department of the Neurosurgery Institute of the National Academy of Medical Sciences of Ukraine, reagents of Biocom, Russia) according to data in the study by Nicolson et al. [11]. To detect beta-hemolytic Group A *Streptococcus* (*Streptococcus pyogenes*, or rheumatogenic *Streptococcus)*, the study used bacteriological seeding from the oropharyngeal mucosa on a selective nutrient medium (DniproLab Laboratory, Ukraine) or by specific antitoxic immunity in blood serum (antistreptolysin O + antistreptodornase and/or streptococcal antihyaluronidase) (ELISA; MDI Limbach Berlin GmbH, Germany). This procedure was reported in the systematic review by Dop et al. [21]. *Candida albicans* infection was diagnosed based on specific IgM in the blood serum (ELISA; MDI Limbach Berlin GmbH, Germany) according to the study by Hughes and Ashwood [16]. Infections caused by *Mycoplasma* and *Chlamydia pneumonia* were detected based on specific IgM in the blood serum (ELISA, the Synevo laboratory, Ukraine) and the PCR of blood leukocytes (The Neurobiochemistry Department of the Neurosurgery Institute of the National Academy of Medical Sciences of Ukraine, reagents of Biocom, Russia). This procedure corresponds to the data of a clinical study by Nicolson et al. [11]. *Borreliosis* and *Yersiniosis* diagnostic tests involved western blot analysis with simultaneous detection of IgM and IgG in several surface and deep antigens of the pathogens (the Synevo laboratory, Ukraine). These procedures followed the approaches of Kuhn et al. [15] and Binstock [10], respectively. *Toxoplasmosis* was diagnosed based on specific IgM, IgA, and IgG in the blood serum (ELISA, the Synevo laboratory, Ukraine), as shown by Nayeri et al. [17] in a corresponding meta-analysis of randomized controlled clinical trials. Congenital *CMV* neuroinfection was identified retrospectively. This test used the anamnestic studies of the newborn’s blood serum (PCR, The Neurobiochemistry Department of the Neurosurgery Institute of the National Academy of Medical Sciences of Ukraine and other laboratory centers as reported by the child’s parents) according to the clinical study by Sakamoto et al. [22]. The congenital *CMV* neuroinfection test also involved a specific neuroimaging, which revealed a pentad of radiological manifestations (ventriculomegaly, cysts in the temporal lobes, areas of impaired cerebral white matter myelination, hypogenesis or agenesis of the corpus callosum and periventricular calcifications). Neuroimaging was interpreted according to the results of an 18-year longitudinal clinical study by Pinillos-Pisón et al. [12]. The mentioned study addressed neuroimaging diagnostics of congenital *CMV* neuroinfection in children [12]. The signs of postnatal acute temporal necrotic-hemorrhagic *HSV-1/2*-etiology encephalitis were verified according to well-prepared clinical reports published in peer-reviewed medical publications. These publications described *HSV-1/2*-neuroinfection as a cause of the clinical picture of ASD in humans [2] (Fig. 2).

Note to Fig. 2. On the MRI of the child’s brain with ASD in the coronal projection (left) and horizontal projection (right) in FLAIR mode, the cystic-fibrotic transformation of both temporal lobes of the cerebral hemispheres is visualized as a result of the previously experienced partial necrotic-hemorrhagic encephalitis of the temporal lobe during early childhood, caused by HSV-1 (own observation).

Thus, patients in both groups underwent a comprehensive assessment of microbial load according to available data from recent scientific reports. This specific approach implied the simultaneous search for all relevant microbial agents. This strategy could reveal a complete picture of the current infection-related state of each child. Subsequently, it was possible to develop an integral structure of infection in the SG. Previous scientific articles published mainly describe the diagnosis of only some, but not all, actual microbial pathogens. In this case, the data are insufficient to conduct a cumulative analysis of microbial load and reproduce a complete picture of infection in ASD. Accordingly, it is impossible to adequately assess the immuno-microbiological connections, as well as determine their role and place in the pathogenesis of a disease, in particular, regarding immuno-dependent mechanisms of encephalopathy development. Careful research on this issue would open the way to the development of new diagnostic and therapeutic approaches.

Other cofactors, apart from microbial agents and GFCD, were not analyzed in children with ASD in this study.

**Statistical processing** of the material involved comparative and structural analyses. The Shapiro-Wilk test demonstrated the distribution of variants in the variational series. To determine the differences between indicators in the studied groups, the study utilized Student’s parametric t-test with a confidence probability indicator *P* and the nonparametric test – the number of Z characters according to V. Y. Urbach. The differences were probable at р<0.05 and $\text{Z}<{\text{Z}}_{0.05}$. The odds ratio (OR) and the 95% confidence interval (95% CI) showed the associations between the studied indicators. The Z-sign test calculation procedure proceeded as follows: a significant difference between the values was determined when the “null hypothesis” was not confirmed (Z < Z_0.05). ($\text{Z}<{\text{Z}}_{0.05}$). The main stages of calculating the sign test include the following:

1. Identifying trends in the compared groups; Dynamics is denoted by the corresponding signs +, –, =; results without dynamics are excluded from further calculations (=).
2. Calculating the number of observations with positive and negative results.
3. Calculating the number of signs that are less likely to appear.
4. Comparing a smaller number of features (Z-test) with a tabular critical value for the corresponding number of observations.

Microsoft Excel (Redmond, WA) served as a tool for statistical calculations.

## Results

**Fig. 3:**
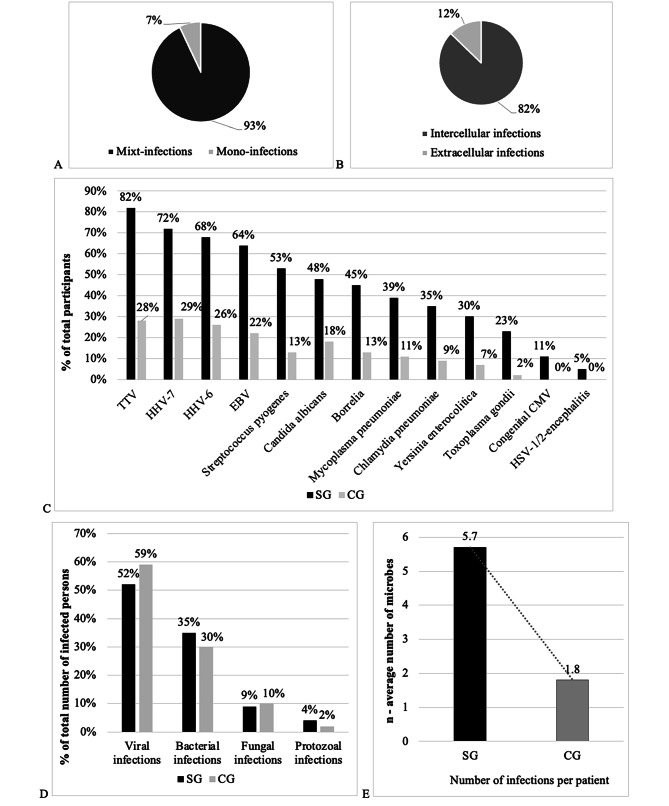
The structural and comparative analyses of data on the studied microbial infections in the SG (*n* = 240) and the CG (*n* = 53)

The structural analysis of the SG for diagnosed microorganisms revealed *TTV* in 196 children (82%), *HHV-7* – in 172 (72%), *HHV-6* – in 162 (68%), *EBV* – 153 (64%), *Streptococcus pyogenes* – 127 in (53%), *Candida albicans* – in 116 (48%), *Borrelia* – in 107 (45%), *Mycoplasma pneumoniae* – in 94 (39%), *Chlamydia pneumoniae* – in 85 (35%), *Yersinia entеrocolitica* – in 71 (30%), *Toxoplasma gondii* – in 54 (23%), congenital *CMV* neuroinfection – 26 (11%), and postnatal *HSV-1/2*-encephalitis – in 11 children (5% of cases) (Fig. 3С). The results of structural analysis of diagnosed microorganisms in the СG revealed *TTV* in 15 (28%), *HHV-7* – in 16 children (29%), *HHV-6 –* in 14 (26%), *EBV* – in 12 (22%), *Streptococcus pyogenes* – in 7 (13%), *Candida albicans* – in 10 (18%), *Borrelia* – in 7 (13%), *Mycoplasma pneumoniae* – in 6 (11%), *Chlamydia pneumoniae* – in 5 (11%), *Yersinia entеrocolitica* – 4 in (7%), *Toxoplasma gondii* – in 1 child (2%). At the same time, the analysis did not find congenital *CMV* neuroinfection and postnatal *HSV-1/2*-encephalitis (Fig. 3С).

A total of 240 children in the SG were diagnosed with 1,374 active infections, which is 5.7 ± 0.36 cases per 1 child on average. It means that 1 patient of the SG had simultaneous laboratory activation signs of 5–6 infectious agents. This fact indicates a large microbial load on a child’s body system (Fig. 3E). In the CG, the mentioned diagnostic methods found 97 cases of microorganisms from the studied list. Thus, there were 1.8 ± 0.12 cases of an infectious agent per 1 child (Fig. 3E). This fact indicates a significantly lower microbial load than in the SG (*p* < p_0.05_; Z < Z_0.05_). Among the CG patients, the tests identified only 0 to 3 infectious agents, which is almost three times less than in the SG (Fig. 3E).

In the SG, there were from 1 to 8 cases of simultaneous identification of different microorganisms in one child. There was not a single child in the SG without at least one infection from the diagnosed list. In the CG, the presence of infectious agents ranged from 0 to 4 per child (*p* < p_0.05_; Z < Z_0.05_). In at least 40% of cases, children from the CG did not show laboratory signs of any studied infection.

Mixed infections in SG largely prevailed over mono-infections (93% against 7% of cases; *p* < p_0.05_; Z < Z_0.05_), in the CG, mono-infections predominated (*p* < p_0.05_; Z < Z_0.05_), (Fig. 3А). Active infections in SG caused by intracellular microorganisms occurred significantly more frequently compared to infections caused by extracellular microbial agents (1131 against 243 cases, or 82 against 18% of the SG; *p* < p_0.05_; Z < Z_0.05_); however, a similar pattern was observed in the CG (p ˃ p_0.05_; Z ˃ Z_0.05_) (Fig. 3В).

Microorganisms of different taxonomic groups occurred with different frequencies. Viral infections in the SG occurred in 720 (52%), bacterial infections – in 484 (35%), fungal infections – in 116 (9%), and parasitic infestations – in 54 children (4% of cases). Thus, viral infections occurred almost twice as often as bacterial infections. Bacterial infections were diagnosed 4 times more often than fungal infections. Finally, fungal infections were diagnosed at least 2 times more often than protozoal infections. However, the structure of SG and CG in terms of the relative proportion of identified viral, bacterial, fungal, and protozoal infections did not differ (Fig. 3D).

As part of mixed infections, microorganisms were combined into arbitrary combinations in different patients without a certain pattern. The only exception was the combination of *EBV* and *Streptococcus pyogenes*. It occurred in almost 90% of cases of rheumatogenic *Streptococcus* among patients in the SG.

There were no gender differences in the structure of the infectious profile and combinations of infections among children in the SG (*p* < p_0.05_; Z < Z_0.05_).

The study found that the microbial load increased with age. In the SG, among children aged under 4 years, the average number of identified microbes per patient was 2.7 cases. At the same time, in children aged 4 to 9 years, there were 6.2 cases, that is, 2.5 times more (*p* < p_0.05_; Z < Z_0.05_). In the CG, there was no age-related increase in the average number of identified microbes of different taxonomic groups (*p* < p_0.05_; Z < Z_0.05_).

The comparative analysis in the studied groups indicated a higher proportion and frequency of all considered infections among the SG patients compared to clinically healthy children in the CG (*p* < p_0.05_; Z < Z_0.05_). In general, the CG children had mono-infections, while the SG children had mixed infections with simultaneous combinations of 5–6 different microorganisms. This result indicates serious differences in the microbial load on the child’s body in the studied groups (*p* < p_0.05_; Z < Z_0.05_) (Fig. 3Е).

Note to Fig. 3. A – Structure of SG based on mono- and mixed infections; B - Structure of SG based on intra- and extracellular infections; C - Structure of SG and CG based on types of microorganisms; D - Structure of SG and CG based on taxonomic groups of microorganisms; E – Average number of microorganisms per patient in SG and CG.

## Discussion

The results of this study indicate that infection with microorganisms from the studied list is an obligate sign of ASD in children with GFCD. In these cases, there is an abnormally large microbial load on the child’s body with a predominance of heterogeneous opportunistic microflora without gender differences. This data suggests the pathological microbial profile in children with ASD associated with GFCD. This microbial profile does not correspond to that in healthy children of the same age.

Thus, according to the priority of detection with these laboratory methods, infectious agents can be arranged in SG in the following order: *TTV* > *HHV-7* > *HHV-6* > *EBV* > *Streptococcus pyogenes* > *Candida albicans* > *Borrelia* > *Mycoplasma pneumoniae* > *Chlamydia pneumoniae* > *Yersinia enterocolitica* > *Toxoplasma gondii* > congenital *CMV* > *HSV-1/2*-encephalitis. This structure of microbial load in patients with ASD differs from healthy children.

Thus, opportunistic viral agents occupy the entire proximal part of the above priority series for identifying microbes on the current list. In turn, bacterial microorganisms occupy the median, while protozoal invasions and marginal forms of viral damage to the CNS cover the distal part. The reasons for the growing microbial burden by age in SG may be an additional infection caused by continuous contact with environmental microorganisms and insufficient elimination of previously obtained microbes, for example, due to weakened immunoresistance.

For most, but not all of the studied infections, the priority of detection in the SG and CG corresponded to each other. This fact suggests the significant prevalence of infectious pathogens in the modern general pediatric population as a leading factor of influence. In turn, some features of immunoresistance in children with ASD associated with GFCD are likely to be a secondary factor of influence.

Research on the immune status in children with ASD can explain such features of microbial load in SG as the predominance of mixed opportunistic intracellular viral infections with low virulence and growth of general microbial burden by age.

For instance, Erbescu et al. [9] conducted a review of clinical trials devoted to the study of abnormalities in the adaptive and innate immunity system in children with ASD. Their results indicate the need to review current ideas about the state of the immune system in autism. It is crucial to strengthen evidence of immune factor imbalance, impaired cytokine status, signs of systemic inflammation, impaired immune tolerance to autoantigens and allergens, persistent intestinal inflammatory disease, and other signs of reduced immunoresistance and immune dysregulation [23, 24].

The predominance of viral opportunistic agents over other infectious agents in SG children can be not only due to the peculiarities of the infectious agent spread in the modern child population. Another reason is the specific immune status of children with ASD. Among their disorders. an important role belongs to the deficiency and/or dysfunction of natural killer cells – innate immunity cells. These cells are essential for controlling viral opportunistic agents in the human body [25]. This feature of the immune status can explain the predominance of intracellular microbes over extracellular ones. Previous studies have already described susceptibility to *HHV-6* and *EBV* in children with ASD [11, 13]. However, the current study is the first to demonstrate that the predominant opportunistic viral agents in such patients are not *HHV-6* and *EBV*, but *TTV* and *HHV-7*. This finding allows for optimizing diagnostic approaches to the assessment of microbial agent infection in children with ASD. Another explanation for the predominance of opportunistic viral microflora in the SG may be GFCD. As the results of recent clinical studies show, an impaired mechanism of herpesviral DNA methylation (which occurs, in particular, in GFCD) can critically affect the reproductive activity of the virus and the resistance of the microorganism to the immune response from the host body [26, 27].

The high frequency of Streptococcus pyogenes cases is consistent with the results of clinical studies among children with ASD. These studies also showed the prevalence of selective deficits in classes and subclasses of immunoglobulins [28], as well as a deficiency of specific anti-polysaccharide antibodies [14], that reduce immunity resistance to rheumatogenic Streptococcus. Non-isolated candida infection can occur due to a phagocyte myeloperoxidase deficiency. The latter is typical among children with ASD since this microbicidal enzyme of phagocytic cells is key in anti-candida protection at the level of innate immunity [29]. In general, microbial-immune issues in children with ASD require further detailed scientific study. Additional data can shed light on important mechanisms of microbial load formation in children with ASD.

The characteristic combination of *EBV* and *Streptocoссus* pyogenes found in SG children is typical. It is associated with allelic combinations in some genetic humoral immune factors, which are critical in the control of these pathogens. Another association is the synergy of these pathogens, leading to the effect of mutual potentiation. It is known that primary deficiency of the IgG3 subclass is a characteristic disorder of the immune status in ASD and causes a decrease in immunity resistance to *EBV* protein antigens. It was found that a combination with a latent deficiency of specific anti-polysaccharide IgG2 is typical for patients with primary deficiency of the IgG3 subclass. It contributes to the development of streptococcal infection, without reducing the total serum concentration of the IgG2 subclass [23]. This allele combination causes simultaneous susceptibility to *EBV* and *Streptococcus pyogenes* in the carrier. On the other hand, streptococcal peptidoglycans activate *EBV* from the latent state in infected cells of the lymphoblastoid line of the tonsils. This process implies the effect of TLR-2 on the innate immunity of the child. Reactivated *EBV*, in turn, through the induction of neutropenia and a decrease in the phagocytic activity of leukocytes, creates favorable conditions for the development of Streptococcus [30].

The proportion and frequency of infections caused by microorganisms of different taxonomic groups in children with ASD associated with GFCD were not the same. In practical contexts, all microorganisms of the studied microbial profile can be divided into 4 groups according to the frequency of their detection. This classification will rationalize, optimize, and stratify diagnostic approaches to the assessment of microbial load in clinical practice. *ТТV*, *HHV-7*, and *HHV-6* comprise Group I of the priority microbes that occur most often. Accordingly, Group I should be the first stage of the diagnostic search and examination. *EBV*, Streptococcus pyogenes, *Candida albicans*, and *Воrrelia* occur with an average frequency and belong to Group II of important microbes. The identification of Group II should be a mandatory component of the further diagnostic process. *Mycoplasma pneumoniae*, *Chlamydia pneumoniae*, and *Yersinia enterocolitica* occur only in every third patient. They are included in Group III of rare microbes. If necessary, Group III can be identified at the last stage of diagnosis. Finally, congenital *CMV* neuroinfection and postnatal *HSV-1/2* encephalitis are rare forms of disorders. These infections comprise Group IV and can be included in supplementary diagnostic searches in the case of relevant clinical and radiological signs.

The microbial profile of the SG children was dominated by the microorganisms currently considered the most typical triggers of immune-dependent complications, including immune-mediated disorders of the central nervous system [18, 20]. Therefore, it is necessary to identify microbial agents in children with ASD. This procedure may be important not only for assessing overall health but also for finding microbial pathways for the formation of encephalopathy. The latter may underlie the children’s existing neuropsychiatric disorders. Al-Beltagi et al. [2] have recently conducted a review of clinical studies addressing the connections between ASD and viral infections. The authors reported a so-called bi-mutual connection between the studied factors. They found that viral infections in early childhood contribute to clinical manifestations of ASD. At the same time, children with ASD demonstrate a higher frequency of viral infections than in the general pediatric population. The current study revealed that microorganisms can affect the state of the nervous system in children with ASD in direct and indirect ways. At the same time, these microorganisms use fundamentally different mechanisms of damage to the brain parenchyma. The direct ways of microbe-induced cerebral damage include infectious encephalitis, which caused the ASD phenotype development [2], as well as some forms of neurodegeneration, such as temporal median sclerosis [31]. The latter occurs in children with ASD [29] and is known to be a result of *HHV-6* [32]. Moreover, temporal median sclerosis probably participates in the pathogenesis of ASD and comorbid epileptic syndrome. Indirect pathways of micro-induced brain damage include allergic [19], immuno-inflammatory [33], and autoimmune mechanisms [9]. These mechanisms correspond to the well-known signs of allergic brain damage [34], diffuse microglial inflammation [1], autoimmune encephalitis [35], and/or demyelinating disorders [36] typical for children with ASD. Among autoimmune lesions, ASD is characterized by the emergence of both antineuronal [37] and antimyelin [36, 38] autoimmunity. This process can refer to microorganisms of the outlined profile acting as triggers for disruption of immune tolerance to brain autoantigens.

Bouboulis and Mast [37] proposed the term “infection-induced autoimmune encephalopathy” to refer to immune-dependent forms of CNS disorders in children with ASD associated with microbial triggers. This concept is broader than PANDAS, an autoimmune CNS disorder associated with rheumatogenic *Streptococcus* [21]. PANDAS was found in some SG patients who showed signs of infection caused by Streptococcus pyogenes.

The connection between GFCD and microbial factors also warrants a separate discussion. A potential mechanism for such a connection could be the negative impact of biochemical disruptions induced by GFCD on the immune system of children with ASD [8]. Also, it is crucial to consider that the methylation process mediated by GFCD influences the gene expression of opportunistic microorganisms inhabiting the human body. Conversely, microorganisms can affect the methylation of specific DNA regions in humans, creating favorable conditions for their parasitic existence. For instance, Engdahl E. et al. demonstrated that HHV-6 is capable of inducing hypomethylation of the 17p13.3 region, correlating with enhanced expression of the viral genome and the phenomenon of viral integration [39].

**Limitations of the study**. Limitations of this study include the absence of randomization and placebo control, as well as its monocentric, monoethnic, and monoracial nature.

## Conclusions

The results of this controlled clinical trial are the first step towards generalizing and systematizing knowledge about microbial infection in children with ASD associated with GFCD. It can become the basis for a microbial concept in autism. This study identified and characterized specific abnormal microbial spectrum, including infection with 13 microorganisms of different taxonomic groups with a large microbial load on the child’s body. The trial revealed the predominance of mixed infections over mono-infections, opportunistic agents – over pathogenic, intracellular over extracellular, viral – over bacterial and fungal, and postnatal infections – over congenital with the formation of arbitrary combinations. The only exception is the combination of EBV and Streptococcus pyogenes. Microbial load increases with age without gender differences. The question remains open whether the considered list of characteristic microorganisms is exhaustive, or whether it requires expanding in further research. This microbial spectrum indicates problems in the somatic health of children with ASD associated with GFCD, including the state of the immune system. At the same time, it can be the subject of further study in the search for potential microbe-dependent mechanisms of encephalopathy development. The latter may underlie the formation of both the ASD phenotype and some comorbid neuropsychiatric syndromes in children. The identified microorganisms of the spectrum can be the objects of therapeutic interventions in research on the effect of antimicrobial therapy on the general health of children with ASD. These interventions can give insights into the severity of mental activity disorder symptoms and potential microbe-induced pathways of damage to the nervous system.

When planning further clinical investigations, it is essential to consider the need for verifying the relevance of the identified microbial spectrum in trials with a larger number of participants, incorporating randomization and placebo control. It is advisable to focus attention on exploring the mechanisms through which microorganisms of a specified profile may influence the severity of ASD manifestations and comorbid syndromes, as well as modify the outcomes of the condition. The exploration of antimicrobial agents for treating infectious processes in children with ASD, along with an examination of the potential impact of microbe elimination on the severity of clinical status and the pace of psychomental development, appears to be a promising research direction.

## Data Availability

The datasets generated during and/or analysed during the current study are not publicly available due to privacy and ethical restrictions but are available from the corresponding author on reasonable request.

## Abbreviations

ASD: Autism spectrum disorders
CG: The control group
CI: Confidence interval
CNS: Central nervous system
EBV: Epstein-Barr virus
GFCD: Genetic folate cycle deficiency
NMDA: N-methyl-D-aspartate
OR: Odds ratio
SG: The study group
